# The Social and Material Life of Antimicrobial Clay: Exploring Antimicrobial Resistance, Medicines' Materiality, and Medicines Optimization

**DOI:** 10.3389/fsoc.2020.00026

**Published:** 2020-04-30

**Authors:** Kimberly Jamie, Gary Sharples

**Affiliations:** ^1^Department of Sociology, Durham University, Durham, United Kingdom; ^2^Department of Biosciences, Durham University, Durham, United Kingdom

**Keywords:** clay, materiality, optimization, social life, geophagy, value, laboratory life, post-colonial theory

## Abstract

While sociologists have made significant theoretical contributions to the antimicrobial resistance (AMR) debate, little attention has been given to the antimicrobial products themselves. Here we advocate a significant new direction which centers on the social and material life of antimicrobials, specifically on what they are made from and how this affects their use. This focus is timely because, in the context of declining efficacy of biomedical antibiotics, diverse materials are increasingly taking center stage in research and drug discovery as potential agents for new antimicrobial treatments. Of particular significance are natural antimicrobials, such as plants, honey and clay, whose antimicrobial potential is well-documented and which are increasingly moving into mainstream antimicrobial research. Alongside this biomedical focus, we suggest that the *social and material lives* of these antimicrobial materials require attention to (i) highlight the ways they have been, and continue to be, used in diverse cultures globally, (ii) explore ways we might theorize these materials within wider AMR debates, and (iii) examine the impact of antimicrobials' materiality on their use by patients. This article takes the example of clay, whose antimicrobial properties are well-established and which has been used to treat wounds and gastrointestinal problems for millennia. We first locate clay as an exemplar of a wider shift toward natural products drug discovery in pharmaceutical science and antimicrobial research. We then offer a number of theoretical “ways in” for sociologists to begin making sense of clay as it comes under the western biomedical gaze. We map these conceptual lenses on to clay's physical and symbolic mobility from its use in the global south into western biomedical research and commercialization. We particularly concentrate on post-colonial theory as a means to understand clay's movement from global south to north; laboratory studies to examine its symbolic transformation to a black-boxed antimicrobial artifact; and valuation practices as a lens to capture its movement from the margins to the mainstream. We finish by reflecting on the importance of materiality in addressing optimal use of medicines and by advocating an interdisciplinary approach to AMR which positions sociology as a key contributor to AMR solutions.

## Introduction

This paper argues for a significant new direction in sociological approaches to antimicrobial resistance (AMR) which focuses on the materiality of antimicrobial artifacts. While social scientists have made significant theoretical contributions to understanding AMR, as well as its framing and responses to it [see Macintyre (2014), Wood (2016), and Will (2018) for overviews], limited attention has been given to antimicrobial products *themselves* and how their materiality (i.e., what they are made from, what they look like, how they are produced) may influence their use. This is despite a recent “materiality turn” in the social sciences (see Pinch and Swedberg, 2008) and the well-established tradition, particularly in science and technology studies (STS), of centralizing non-human artifacts; Bruno Latour, after all, reminds us to “follow the actors” (Latour, 2005, p. 237).

Such a focus on antimicrobials' materiality is important because, in the context of the declining efficacy of existing antimicrobial medicines, diverse materials are beginning to take center stage in research and drug discovery as potential agents for new treatments (Newman, 2019). Of particular significance are natural products such as honey, plants, and clay whose antimicrobial potential is well-known and are therefore prime candidates for new antimicrobial drug discovery programmes (see McLoone et al., 2016 for a review of honey). The application of computational biological approaches and modern high throughput screening techniques to uncover nature's “treasure trove” Davies (2011, p. 5) opens up the potential for a fresh generation of antimicrobials derived from natural products (Thomford et al., 2018). Alongside this biomedical focus on diverse materials, we suggest that sociologists are best placed to make sense of the *social and material lives* of these natural antimicrobials, particularly to locate their materiality within discussions of optimizing their use.

In this article we take the example of clay, whose antimicrobial properties are well-documented and which has been used for centuries to treat wound infections and gastrointestinal problems (e.g., Williams and Haydel, 2010; Williams, 2019). As an exemplar of the accession of natural products into western antimicrobial research, we suggest that clay provides a fruitful lens to explore the shifting material life of antimicrobials. We offer a number of theoretical “ways in” to this exploration, which we map onto the physical and symbolic mobility of clay from its use in the global south into western biomedical research. These “ways in” are not intended to be comprehensive; we offer only brief overviews of each theoretical approach and pose questions and suggestions, rather than answers, as to how they may be useful in understanding natural product drug discovery approaches to antimicrobials. We also deliberately avoid over-synthesizing these “ways in” as we do not wish to present an instructional schema for researchers approaching clay sociologically. The intention, then, is to set an agenda for a novel approach to AMR which centers on antimicrobial products and to demonstrate its theoretical feasibility.

First, we offer an overview of the shift toward natural antimicrobials before we hone in on clay and examine the ways that social scientists have previously made sense of medicinal applications of clay, primarily through its ingestion. We then suggest a number of theoretical “ways in” for sociologists to begin thinking specifically about the place of clay in the antimicrobial landscape, but more widely about the materiality of diverse antimicrobial products and what this might mean for their use by practitioners and patients. We then consider what insights can be learned for optimizing antibiotic use through this focus on materiality. Finally, we argue for an interdisciplinary approach to AMR in which sociologists collaborate not just with our closest disciplinary neighbors, but across the natural and physical sciences boundaries in order to position sociology as an important contributor to AMR policy and practice solutions (see Will, 2018).

## Antimicorbial Resistance and the Shift Toward Natural Products Drug Discovery

### Antimicrobial Resistance

AMR refers to changes in pathogenic (that is disease-causing) microorganisms (viruses, bacteria, fungi, or protozoa) that allow them to acquire resistance to existing medication or treatment regimes. Promoted by inappropriate and excessive use of antimicrobials in human and veterinary medicine, AMR poses a significant global public health problem as common infections become increasingly challenging to treat and previously routine surgical procedures become potentially hazardous (World Health Organization, 2014). Coupled with this, few new antimicrobial drugs have been discovered or developed in recent years while history has demonstrated that further evolution of resistance is inevitable (Rodríguez-Rojas et al., 2013). This has created an “antimicrobial perfect storm” (Broom et al., 2014, p. 81) which the United Nations (2019, p. 1) suggests will have a “disastrous impact within a generation.” In the UK, a recent government report predicted that AMR is likely to overtake cancer as the leading cause of death over the next 30 years (O'Neill, 2016), while the World Health Organization (2014, p. 19) estimated that the current $21–34 billion/year cost of AMR to the US health system will escalate as drug resistance increases. de Sosa et al. (2010) note that the impact of AMR is likely to be more extreme in developing countries where a higher infectious disease burden and precarious financial circumstances prevent the rapid development and deployment of new treatment agents.

### Natural Products Drug Discovery

In the context of the declining efficacy of existing antimicrobials, biomedical researchers are turning to increasingly innovative methods, approaches and materials to identify new agents. A key aspect of this is “natural products drug discovery” where researchers look to natural materials for their therapeutic potential; in other words, science “revert[s] to ‘nature’ for answers” (Thomford et al., 2018, p. 1). While this movement toward natural products represents a shift in contemporary drug discovery practice, the natural world is not uncharted territory for pharmaceutical research and drug discovery (Mandal et al., 2018). Rather, a significant number of well-established biomedical therapies are derived from natural products, including quinine (antimalarial from the bark of the Cinchona tree), codeine (painkiller from poppies), and Taxol (cancer therapy from the Pacific yew tree). Indeed, natural products drug discovery as an approach within the pharmaceutical industry had a “golden age” in the 1950s and 1960s during which time the US Department of Agriculture undertook a specific programme of plant extract collection and screening (Cragg and Newman, 2015) and a significant number of naturally-derived pharmaceutical products were brought to market (Shen, 2015, p. 1297). This heyday of natural product drug discovery was tied up with what has been called the “golden age of antibiotics” during the middle decades of the twentieth century when the discovery of new antibiotics coincided, in the UK, with improvements in social housing and the introduction of the National Health Service. In this new healthcare and public health milieu, antibiotics were positioned as wonder-drugs heralding the end of infectious disease (see Burnett, 1953). It should be noted that the majority of antibiotics discovered during this period, and subsequently, originated as natural products (Newman, 2019).

In the latter half of the twentieth century, however, many pharmaceutical companies scaled back their focus on natural products as high throughput screening and novel synthesis methods enabled the creation of large libraries of synthetic chemicals (Shen, 2015). Moreover, accessing, harvesting, and growing natural products involves navigating diverse, and often competing, political, environmental and financial interests, potentially making naturally-derived products practically and financially unviable [see Goodman and Walsh's (2001) overview of the political landscape surrounding Taxol]. As such, natural products drug discovery largely fell out of fashion as pharmaceutical companies favored the use of synthetic compounds in drug manufacturing. Antibiotic development is also faced with significant difficulties associated with passing drug trial hurdles and the perceived and genuine lack of profitability inherent in short-term treatments.

More recently, however, in the context of the AMR crisis, natural products have begun to (re)take center stage as companies explore new avenues for potential antimicrobial drug candidates (Cragg and Newman, 2013). In this context, the natural world has been described as an “endless frontier” (Li and Vederas, 2009) and an “inexhaustible” (Davies, 2011, p. 5) source of potential candidates for new antimicrobial therapies (see Brown and Wright, 2016). Coupled with advances in microbial genomics, bioinformatics, and synthetic biology (see Thomford et al., 2018), it has been suggested that drug discovery is undergoing a “renaissance…inspired by natural products” (Harvey, 2007, p. 480) where “we are surely entering a new golden age of natural products drug discovery” (Shen, 2015, p. 1297).

The therapeutic use of natural products is, of course, not limited to pharmaceutical medicines. Natural healing products on general sale such as Aloe Vera, Manuka honey and Echinacea have been used as antimicrobial treatments for centuries and are growing in popularity in the west. Sociologists have theorized this increased use of natural products (mostly subsumed under the umbrella of “complementary and alternative medicines”) as a result of their commercialization (Collyer, 2004), increased skepticism toward biomedicine, dissatisfaction with traditional doctor/patient relationships and a proliferation of discourses of holism in health [see Gale (2014) for an overview]. For Carter et al. (2016), the increased use of these natural products and, in many cases, their adoption as potential antimicrobial drug candidates by pharmaceutical companies, repositions them from alternative, marginal therapies into the mainstream.

### The Case of Clay

One such example is clay (or more specifically minerals found naturally in clay), whose antimicrobial properties are well-documented (e.g., Williams, 2019) and which has been used in various therapeutic forms since the earliest human civilizations (Hosseinkhani et al., 2017). While therapeutic clay use has a long history, the contemporary biomedical story of antimicrobial clay minerals begins in 2002 when French humanitarian worker Line Brunet de Courssou approached the World Health Organization with a series of case studies in which she had used clay, specifically French green clay, to treat Buruli ulcer (a necrotising soft tissue disease caused by *Mycobacterium ulcerans* and where treatment involves combined antibiotic therapy and often surgery, including amputation) in Côte d'Ivoire (Williams and Haydel, 2010). In her report, Brunet de Courssou suggested that clay may provide an effective way to treat bacterial skin infections commonly found in Africa and requested financial support to further research the area. Her application for funding was unsuccessful but nonetheless prompted scientists in North America to pursue the antimicrobial potential of clay and its mode of action. Williams and Haydel (2010, p. 746), for example, state that Brunet de Courssou's findings “were the stimuli for our research into the healing mechanism of clays” and, in the same paper, thank Brunt de Courssou for bringing antibacterial clays to their attention.

In the years following Brunet de Courssou's report, western researchers began to analyse the mineralogical properties of the clays used in her work and to ask, more generally, “what makes… clay antibacterial?” (Williams et al., 2011). In other words, while the healing properties of clay have been known around the world for thousands of years, researchers began to investigate the biochemical and mineralogical basis for their therapeutic benefits. In more recent years, as AMR has loomed larger as a global health threat, the antimicrobial potential of clay minerals has been centralized as part of the so-called “new golden age of natural products drug discovery” (Shen, 2015, p. 1,297) and the antimicrobial properties of clay are beginning to gain traction in drug discovery science (e.g., Morrison et al., 2016). Within this, clay-based topical therapies have been shown to be effective in treating skin conditions, including necrotising fasciitis, as noted above (Williams et al., 2004), open wounds (Sirousazar et al., 2011), and acne (Toombs, 2005). Detailed analysis of clays and their impact on bacterial survival has led to the suggestion that the aluminum and iron content of clays is the toxic component (i.e., what kills bacteria), probably as a result of localized release of reactive oxygen species at the bacterial surface (Morrison et al., 2016; Wang et al., 2017; Zarate-Reyes et al., 2018). The antibacterial action of clays has been further evidenced in other studies. Considerable work has been undertaken to characterize the chemical and physical properties of clays responsible for antibacterial activity (Williams, 2017, 2019). The efficacy of clay against pathogenic bacterial biofilms has also been confirmed (Caflisch et al., 2018) and antibacterial clays have been found to reduce drug-resistant *Staphylococcus aureus* infection and inflammation in wound infections in mice (Otto et al., 2016). As such, there is a clear, and continually emerging, body of scientific evidence that clay is an effective antimicrobial, and Williams (2019, p. 7) suggests that in the context of a growing antimicrobial crisis “mimicking the antibacterial mechanisms exhibited by natural clays could be advantageous in the development of new antimicrobial agents.”

While the story of clay, and other natural antimicrobials, in contemporary drug discovery is interesting in itself, in this article we are concerned with how sociologists might make sense of this shift in drug discovery science and theoretically locate natural antimicrobials within the wider AMR landscape. To begin to address this, for the remainder of this paper we highlight clay as an example of the (re)entrance of natural materials into AMR research and drug discovery. Through this example, we show that the reinvigoration of natural products drug discovery requires a collaborative, interdisciplinary approach which locates the social and material life of natural antimicrobial products alongside their biochemical and antimicrobial potential. Such an interdisciplinary approach not only makes visible the uses of these products across cultural contexts but also, by centralizing the materiality of natural antimicrobials, has potential impacts for optimizing antimicrobial use.

To do this, we suggest theoretical “ways in” through which sociologists might explore the material life of clay and map these onto its mobility. Here we mean both the physical movement of clay (from the global south to the global north, from poor countries to rich ones, from black to white bodies, and from natural contexts to research laboratories) and its symbolic mobility from the margins of western medicine into the mainstream. We do not intend to provide a comprehensive or instructional account of how to explore medicinal clay and other antimicrobials. There are undoubtedly other theoretical framings left unexplored here which could be effectively mobilized to theorize the use of clay, and other natural products, in antimicrobial research. Rather, the “ways in” we propose are intended as heuristic devices to stimulate a novel sociological discussion of, and focus upon, the materiality of antimicrobial artifacts. While we focus on the case of clay specifically, the approaches we outline below are applicable to other materials emerging from natural products antimicrobial discovery and, throughout, we provide examples of this wider applicability.

## Sociological “Ways in” to Antimicrobial Clay

### Existing Social Science Research on Clay: Geophagy

Unsurprisingly, clay has not been prominent on the radar of medical sociologists. That is not to say, however, that clay has wholly escaped the attention of social scientists; anthropologists and geographers have had a sustained interest in clay's medicinal uses, particularly the practice of geophagy, which is the deliberate eating of soil, earth, or clay. Ingestion of clay has a number of proposed benefits, notably through its mineral content serving as a nutritional supplement and its absorptive properties in detoxification and lining the gut to settle gastrointestinal infections (potentially also as an antimicrobial), and is common across the global south and east (Henry and Cring, 2013, p. 181). The practice of geophagy, broadly speaking, has been conceptualized in one of two ways (see Henry and Matthews Kwong, 2003). It is either (i) pathologized as a form of “pica,” that is “compulsive eating of non-food substances” (Walker et al., 1997, p. 280), or (ii) understood as a routine part of everyday nutritional life and foodways (Loveland et al., 1989). While medicine (particularly psychiatry and public health) has focused on the neurological causes of geophagy, its negative health consequences and possible treatments, social scientists have foregrounded geophagy's cultural-locatedness and ordinariness in many cultures and communities globally.

Central to this tension, following Douglas (1966), is the distinction between “food” and “non-food” (Gonzalez Turmo, 2009) and the positioning of soil within it. In the contemporary west, soil is understood as “a polluting non-food” which is “too natural to be acceptable” (Henry and Matthews Kwong, 2003, p. 361–2). Consuming soil in this context is, therefore, highly stigmatized (Forsyth and Benoit, 1989) and associated with groups already viewed with a degree of “otherness” such as women (Allport, 2002), children (Young, 2011) and poor, rural, black populations of the Southern US states (Frate, 1984).

Conversely, social science approaches to geophagy have highlighted clay's legitimacy as a foodstuff and a routine part of health and nutritional practices in, among other places, Nigeria (Vermeer, 1966), Ghana (Vermeer, 1971; Hunter, 1973), Kenya (Geissler et al., 1997), and the Southern USA (Hertz, 1947; Frate, 1984; Forsyth and Benoit, 1989). For Henry and Cring (2013, p. 181), geophagy's embeddedness within the ebbs and flows of everyday life brings with it knowledges and practices, particularly around selecting and preparing clay, which “bring it into culture.” In other words, skilled knowledge of choosing which clays are edible and how to prepare them correctly for consumption brings geophagy out from hidden sub-cultural corners and into the mainstream. In his colonial explorations of South America, Von Humboldt (1872, p. 495) noted that people do not “eat every kind of clay indifferently” but, rather, select specific types of clay for eating based on smell, taste and texture (Geissler et al., 1997) or location (Hertz, 1947). In the Southern USA, Frate (1984, p. 35) compared the selection of edible soil to the selection of wine, with both gaining “a reputation over the years” and becoming known for their provenance (also Forsyth and Benoit, 1989, p. 66).

Across both medical and social science approaches to geophagy, pregnant women occupy a particularly prominent position as wider pica behaviors are associated with pregnancy cravings. Reported rates of geophagy among pregnant women vary considerably from 0.2% in Denmark (Mikkelsen et al., 2006) to 92.5% in Nigeria (Izugbara, 2003). Medical approaches to geophagy in pregnancy focus on the risks of helminth infection, lead poisoning, dental injury, and gastrointestinal complications (Ezzeddin et al., 2015). Thus, the predominant medical discourse has constructed geophagy and pica in pregnancy as a “dangerous form of self-injurious behavior” (Williams and McAdam, 2012, p. 2050) and focused on prevention, particularly through educational programmes for women in the global south.

Conversely, social scientists have pointed out that geophagy in pregnancy in non-western settings is tied up with wider cultural practices, beliefs, and “symbolic links between people, fertility, good health, and ancestral blessings” (Njiru et al., 2011, p. 455). Geissler et al. (1998) and Izugbara (2003) also highlight the sacredness of pregnancy-related geophagy and its associations with fertility deities and the perceived life-giving forces of the earth. As such, as a highly gendered practice where history, place, culture, family, gods, and female bodies meet, the medical approach of simply educating pregnant women against geophagy becomes complex (Corbett et al., 2003). Researchers have demonstrated that treating indigenous people, places, and cultures as *tabula rasa* onto which western biomedical messages can be inscribed ignores existing cultural practices, and can lead to significant, and potentially harmful, distortions of public health messages (e.g., Williams-Blangero et al., 1998).

Clay has, then, enjoyed some prominence in social science literature albeit amalgamated with other types of soil and earth and examined almost wholly through the lens of geophagy by anthropologists and geographers. There is limited research on what clay-based practices are currently occurring globally as wider medicinal uses of clay only occasionally appear in geophagy research (e.g., Izugbara, 2003, p. 194) and anthropological research on geophagy has slowed considerably since a flurry of activity in the 1970s. While geophagy research provides a useful context and establishes the widespread use of clay as a medicinal antimicrobial artifact, this article advocates a broader sociological investigation into clay as it moves into mainstream biomedical research as part of a focus on natural products antimicrobial drug discovery. As clay and other natural antimicrobials increasingly take center stage in antimicrobial drug research, credible theoretical lenses to their social, and material lives will be vital for ensuring sociologists are included in the policy, practice, and research conversation. We now turn to propose three theoretical “ways in” to understanding clay as an antimicrobial agent which we map onto the physical and symbolic mobility of clay into, within, and out of western biomedical laboratories.

### Way in 1: Clay in Context: Post-colonial Approaches

A post-colonial lens is perhaps the most logical entry point into a sociological analysis of medicinal clay as it provides a way to theorize clay, and its use, in its de-westernized context prior to its movement and adoption into western biomedicine.

In short, post-colonialism, as a set of intersecting theoretical approaches, is concerned with the legacies of colonial ideologies and power and the ways in which contemporary global economics, politics and culture are rooted in colonial projects. Said's (1978) seminal text *Orientalism* is pertinent here. In it, he outlines the ways that western powers, through centuries of colonial rule, came to define indigenous people and practices in the global south and east [what Hall (1996) calls the “non-west”] as oriental “other,” engaging in and driven by “savage,” strange and “primitive” beliefs and practices.

Post-colonial theories have usefully been applied to understand the history of medicine (see Anderson, 1998) and science (see Seth, 2009) where researchers have, among other foci, drawn attention to colonialism's consistent devaluing of traditional practices and knowledge in favor of a model where western medicine and science were understood as “gifts” to colonies (Seth, 2009, p. 373). Philosopher Lévy-Bruhl's work on “the primitive” is a striking example of the naturalization of European medical and scientific superiority which post-colonial scholars seek to untangle. In it, Lévy-Bruhl consistently utilizes anecdotes of behavior from diverse contexts to reproduce a distinction between “primitives” and the “civilized” world, confirming the superiority of Europe and entrenching the notion that colonized nations “are simply different from us” [see Bernasconi (2005), p. 231–22 for an overview].

Similar devaluing of indigenous cultures is echoed elsewhere in relation to medical practice specifically. In her overview of British colonial perceptions of Indian and Burmese medicine, Edwards (2010, p. 28) notes that in the late nineteenth century, traditional Indian medicine was dismissed as “despicable quackery” despite some “western” practices, such as inoculation against smallpox, having been practiced for centuries before the British arrived. In Zimbabwe, this valorization of traditional medicine went further where, under the Witchcraft Suppression Act (1899), the majority of traditional medical healers, practices, materials, medicines and objects were criminalized for being non-scientific and dangerous (Mawere, 2014).

By centralizing and problematizing this process of devaluing traditional medical practices, post-colonial approaches offer a useful lens for understanding the ways medicinal clay is positioned within modern, western medical practice. This can clearly be seen in the case of geophagy where colonial gazes “are still active today” in framing this practice as a form of pica (Henry and Cring, 2013, p. 186). Beyond this, however, post-colonialism can help us question some of the fundamental constructs underpinning the story of medicinal clay's emergence into western biomedicine, most notably claims about its newness and the infrastructure through which it physically moves to the west.

First, as we have mentioned above, the use of clay for antimicrobial purposes is not novel; clay has had a sustained prominence throughout medical history. While most contemporary biomedical papers on clay outline this longer history as context for a biomedical framing, this in itself is part of the problem—medicinal clay use is constructed, through this narrative, as an historical artifact, and a set of knowledges circulating around the great ancient civilizations, rather than a practice which is *still* continuing in formerly colonized spaces. Williams' (2019) recent overview of clay's historic and continued use globally, for example, opens with accounts of clay use by the earliest humans and its importance in ancient Greek and Roman medicine while devoting almost no space to the *current* use of clay in the global south.

This kind of positioning of natural antimicrobials invisibilizes medical knowledge and practice from contexts where natural products form the basis of much traditional and contemporary medical practice. In the case of plants, the World Health Organization (2005) suggests that 80% of the world's population uses traditional, plant-derived medicine as primary health care. Moreover, in a report on the *State of the World's Plants*, Willis (2017, p. 22) notes that at least 28,187 species are recorded as being of medicinal use, mostly in rural areas of the global south where traditional medicine is accessible and affordable, and often trusted more than western pharmaceuticals.

Despite this, the contemporary relevance of medicinal clay is located almost wholly within the frame of contemporary, western medicine. The “story” of clay's emergence into medicine (much as we have told it above) starts at the point of its mobility into the western biomedical gaze through Brunet de Courssou's work and constructs a future which is entangled with western biomedical agendas (AMR, safety concerns), technologies (screening, analytics), and practices (patenting, commercializing). Taking a post-colonial approach helps to elaborate on the de-westernized story of medicinal clay, locating it within non-western knowledge assemblages, markets and traditions.

Post-colonial approaches can also help us attend to the implications of clay's physical mobility as it moves from its natural (potentially sacred) contexts in the global south into western biomedical research spaces. Here, the notion of “bioprospecting” becomes useful. For Hayden (2003, p. 1) this refers to “corporate drug development based on medicinal plants, traditional knowledge and microbes culled from the ‘biodiversity-rich’ regions of the globe.” Schiebinger (2004) has related the modern practice of bioprospecting to the actions of early European colonialists who exploited plant sources in the global south in the name of botany and medical science. While contemporary bioprospecting legislation requires corporations to remunerate indigenous populations for the exploitation of their land and resources, this model nonetheless naturalizes the trade of goods from south to north, poor to rich, and prioritizes western scientific and corporate development. Most of the existing work on bioprospecting looks specifically at the case of plants but, as clay begins to gain traction as a biomedical substance, this scope could be broadened to interrogate where the clay in western biomedical laboratories has come from, and through what means it arrived.

### Way in 2: Movement Around and Out of Science: Revisiting Laboratory Life

Once clay has, then, moved from the global south into the western biomedical gaze, it is subject to scientific work where the evidence of its antimicrobial potential is established. Given this, our next “way in” to understanding antimicrobials derived from natural products is slightly different in proposing both a theoretical *and* methodological approach. In particular, we suggest a return to “laboratory life” to capture the ways that “the daily activities of working scientists lead to the construction of scientific facts” (Latour and Woolgar, 1979, p. 40). We use the phrase “return to” deliberately because, as Doing (2008) points out, following a flurry of publications during the 1970s and 1980s, few laboratory studies have actually emerged out of STS scholarship, despite their foundational impact on the field. Given this, Doing (2008, p. 281) goes further and calls for “a reengagement between ethnographic work in laboratories and the now established field of STS.” We suggest that the (re)emerging field of natural product drug discovery would provide an excellent site for such a reengagement and would illuminate the ways in which the antimicrobial potential of clay is brought into being.

Briefly, classical laboratory studies were concerned with how scientific facts are produced interactionally, through everyday scientific experimentation, discussion, technologies and negotiation. As Knorr Cetina (1995, p. 141) argues, the mission of these studies was to show the “process of knowledge production as ‘constructive’ rather than descriptive.” Such a focus represented a shift from demarcationist philosophy such as that of Karl Popper who argued that a distinct demarcation between science and non-science could exist. In contrast, laboratory studies were, and still are, concerned with the production of scientific knowledge *in situ* and uncovering the messiness of scientific practice which is invisibilized in publications where scientific facts and methods are presented as fixed and logical (Knorr Cetina, 1981). More recently, in his research on pharmaceutical company chemical laboratories, Barry (2005) shows that molecules are not “discovered” but, rather, “invented” as “informed materials” through laboratory work wherein the material structure becomes richer and better-known through the compilation of information and data. This is echoed by Hardon and Sanabria (2017, p. 118) who suggest that “there is no pure (pharmaceutical) object that precedes its socialization and interpretation.” Laboratory studies are, then, concerned with uncovering the processes of this socialization and interpretation.

While research in this tradition primarily focused on the microsocial action of everyday work within specific laboratories, Fujimura (1987) usefully demonstrated the constraints and influences on scientific work from “outside” of individual laboratories, such as from regulators, sponsors and industries. In this sense, the construction of scientific facts is not just contingent upon everyday work in the laboratory but an alignment of local (the necessary laboratory tasks are doable), institutional (these tasks are feasible within a given laboratory space), and wider field (the research is viewed as worthwhile by the broader scientific community) concerns and constraints (Fujimura, 1987).

Given we are advocating a directional shift toward the materiality of natural antimicrobials, the laboratories in which this materiality is produced is a logical research site for sociologists. Such a focus would illuminate the ways in which clay materials are inscribed with antimicrobial potential and how this potential is represented to the wider scientific community through publications (see Latour and Woolgar, 1979). In the case of clay, scientists use microbiological and biochemical techniques to probe the susceptibility of microorganisms (e.g., minimum inhibitory concentrations, viability assays) and define the mechanism of action (e.g., structural and elemental analysis of the clays themselves coupled with molecular effects on microbial constituents, such as lipids, proteins and nucleic acids) (see Williams, 2019). These methodologies and technologies act as an “inscription device” to “transform [the] material substance into a figure or diagram” (Latour and Woolgar, 1979, p. 51), which is then used to tell the story of the antimicrobial action of a particular clay. A sociological investigation into the inner workings of this process would be valuable but so too would an analysis of the entanglements of actors, both human (e.g., scientists, marketers, funding panels) and non-human (e.g., technologies, images, research agendas). For example, Goodman and Walsh (2001) highlight the case of Taxol obtained from the Pacific yew tree, whereby disharmony in natural antimicrobial science was not limited to members of a particular scientific group but extended to politicians, funders, associated industrial stakeholders (lumber or mining companies) and indigenous populations. This final point, of course, circles back to post-colonial approaches.

A focus on the laboratory life of natural antimicrobials would also usefully go beyond the physical limits of the laboratory to follow clay on its physical and symbolic journey to its scientific facthood in other spaces. In other words, we might usefully ask how is the “fact” of clay's antimicrobial potential constructed, and what happens to this “fact” once it is black-boxed and leaves the laboratory through scientific papers as “evidence.” This focus particularly calls to mind other STS work around the role of hope, promises and expectations. Much of this work is centered on the future-oriented discourses to emerge from the Human Genome Project and its resultant technologies (e.g., Hedgecoe and Martin, 2003). This approach recognizes the social and political life of scientific expectations, acknowledging that these promises shape research agendas and can hold significant power in mobilizing resources at micro, meso and macro levels of science work (Borup et al., 2006). In the case of natural antimicrobials, scientific papers are awash with expectations of their revolutionary potential. In some cases, these promissory discourses have also crossed the rubicon into media reporting which is predictably filled with sensationalized accounts of natural antimicrobials' potential. For example, the Daily Mail (Andrews, 2018) reports the antimicrobial action of Atlantic sponges as “revolutionary,” while *Independent* journalist Rodgers (2007) asks whether clay might be the “new Penicillin.” Research is needed to understand what purpose these promises serve. In particular because as the (re)emergence of natural antimicrobial drug discovery is in its infancy and global attention is increasingly turned toward innovative approaches to AMR, the political potential of these promises is worth unpacking.

### Way in 3: Moving From the Margins to the Mainstream: Developing Clay's Value

As clay physically moves from the global south into western biomedical research spaces and out again as a black-boxed antimicrobial fact, it also shifts symbolically from inert “non-stuff” into an artifact with potential value.

Here we are not talking only about commercial worth but rather value as an entanglement of social, cultural, scientific, medical, and economic value. What we are specifically referring to is clay's movement from the margins of biomedicine (associated with “alternative” medical practices of the non-west) into the mainstream (as a credible biomedical antimicrobial) and the concomitant social legitimization and economic valuation of clay as an artifact or material. In other words, using Saks (1995) power model, as clay moves toward the power structures of science, medicine and healthcare, it attains value and legitimacy and its placement within the category “alternative” becomes ambiguous.

This movement of clay from the margins to the mainstream is partly driven by the changing evidence base around its antimicrobial potential. As clay moves into the western biomedical gaze, evidence about its antimicrobial functionality is increasingly obtained from standardized research practices which are understood as more legitimate than “anecdotal” observations or case studies from the past or from the global south (see Timmermans and Berg, 2003). A sociological exploration of clay would do well to interrogate this evidential shift to analyse the ways that clay's antimicrobial “facthood” comes into being and becomes reified.

Here too the notion of valuation practices can help create a more holistic approach which incorporates, by moving beyond, economics, and evidence. Dussauge et al.'s (2015) recently developed notion of “valuographies” may be helpfully employed to understand the potential antimicrobial value attributed to clay as it moves into the western biomedical gaze. In the concluding chapter of their anthology on value practices, Dussauge et al. (2015, p. 266) call for more research exploring “values in-the-making” in medicine and the life sciences to “examine how certain things come to be considered valuable and desirable” and what the implications are of increased desirability. In one of the anthology's chapters, Löwy (2015), for example, highlights how the increased valuation of prenatal screening for Down Syndrome (by both clinicians and parents) repositioned the test from a niche procedure in high risk cases to a mainstream tool enmeshed in discourses of eugenics. In other words, as the non-essential desirability of prenatal screening increased, it shifted out of specialist obstetric practice and into mainstream pregnancy care carrying with it financial implications for service providers, and increased surveillance of pregnant bodies.

Central to Dussauge et al.'s (2015, p. 7) work is an ambition is to move away from a construction of value *wholly* “revolving around capital and labor” to one in which multiple value forms are commensurable and are dynamically created and recreated in practice. A complementary reading of Garcia-Parpet's (2007) work on the construction of “perfect” markets is useful here to shed light on the social construction of new economic markets. Through a focus on a strawberry auction in Fontaines-de-Sologne, Garcia-Parpet demonstrates that the development of “perfect” markets (see pages 25–26 for an overview) is not solely reliant on financial equilibrium massaged by “invisible hands” of self-interest but is in a constant state of (re)creation through the development of networks, vigilance, and the social identities of the actors involved. In other words, Garcia-Parpet (2007, p. 20) shows that “social factors… [intervene] all across the practical processes of making up this, the purest of ‘economic’ markets.”

Such entanglements of actors, technologies, products and finances (as captured by the valuographies model) are important for understanding the ways in which natural antimicrobials attain both social and economic value; in other words, how they come to be both desirable (i.e., legitimized) and profitable (i.e., have markets created around them). We might, for example, ask how clay is positioned within a scientific research landscape where fundamental research is increasingly eclipsed by research guided by industrial agendas, which are necessarily profit-driven (Quaglione et al., 2014). How, as clay gains mainstream biomedical value, do research questions change from fundamental explorations of how clay works (e.g., Williams et al., 2011) to questions of application, commercialization and increased efficiency of clay-based medical products and practices? Furthermore, how might a “perfect market” as exemplified in Garcia-Parpet's work, develop around clay?

However, clay's increased social and economic value does not just lie in its potential scientific and medical applications, but also in its marketability as a beauty and cosmetic artifact. In recent years, clay has made a rather startling appearance onto the western beauty scene with promises to do things like “clean,” “detoxify” and “renew” in a “natural” and environmentally-conscious way. This entrance of clay into the western beauty landscape has yet to be theorized but the increased value of clay in this space chimes with several existing social science research concerns such as the movement toward ecologically-sound capitalism, the increased appetite for “natural” lifestyles (Edmonds, 2008), and the desire to engage in non-western practices which are seen as “authentic” (Campbell, 2008).

This increased presence of clay in beauty and cosmetic products raises questions about the discourses of “detoxification” and “cleansing” which underpin clay-based beauty products, particularly with respect to what they accomplish and to whom they are addressed. Theoretically, one might put Douglas (1966) to work here to understand the construction of bodily pollutants and read this alongside feminist work which highlights the inscription of gender norms (in this case to be clean, pure, and detoxified) onto female bodies (e.g., Wolf, 1991). This would help us to appreciate, again employing Dussauge et al. (2015) work, how a commercial market (this one focused on beauty and cosmetics) around clay is developing.

## Exploring the Relevance of Materiality for Antibiotic Adherence and Optimization

While research through any, and all, of these “ways in” would be intellectually meaningful, this novel focus on materiality goes beyond theoretical talking points and has implications for optimizing antimicrobial use. Most adherence research to date has been preoccupied with identifying patterns of medicines use based on demographic factors such as age, sex, socio-economic status and ethnicity. These models, however, fail to address how, within complex social worlds, medicines-use decisions are actually formulated, and the nuanced reasons why patients may utilize medicines sub-optimally (Rathbone et al., 2017).

Within social sciences, progress has been made on remedying this rather one-dimensional approach by placing patients' beliefs and wider lifeworlds at the core of analysis, positioning medicines as “socially embedded phenomena” where decisions about use are made within a complex web of relationships, spaces, roles and identities (Cohen et al., 2001). Conrad (1985) calls this “medication practice” and highlights the ways suboptimal medicines use can be a form of control for patients. Others highlight the importance of place and space in patient relationships with medicines (Hodgetts et al., 2011; Dew et al., 2014) and the mobility of clinical categories between spaces Webster, Douglas and Lewis (2009).

Anthropologists have made the most significant leaps in mapping the “social lives of medicines,” highlighting that medicines are more than just chemical *things* and, instead, take on social, economic, and political meanings which can affect the ways they are used (Whyte et al., 2002). In their comprehensive review of recent work in the anthropology of pharmaceuticals, Hardon and Sanabria (2017, p. 118) convincingly outline the ways that “drugs are rendered efficacious in laboratories, therapeutic settings, drug outlets, and everyday lives across regulatory settings.” Refreshing, and relevant here, is that their paper aims to “examine what lies beneath the pharmaceutical object's surface, unpacking the thing” (ibid). Through an overview of the construction of medicines at five key sites in their lifecycle (trials, regulatory frameworks, marketing, care practices, and in individual bodies), they demonstrate that the use of medicines is relational and intertwined with their diverse inscriptions, and part of an on-going, constantly evolving interaction between the identities of patients and medicines themselves (Rathbone et al., 2017).

Despite this, their review falls somewhat short of its promise to burrow beneath the surface of medicines and open up the “thing” (Appadurai, 1986). Inasmuch, they commence their narrative with randomized controlled trials (RCTs), overlooking the underpinning scientific studies during which the material life of the medicinal thing is of central importance. Hardon and Sanabria's (2017) paper is an exemplar of a wider approach wherein medicines themselves (particularly their active ingredients) are essentialised and positioned as black-boxed objects around which practices, values, beliefs, behaviors and identities circulate. In other words, while the focus on social and cultural lives of medicines is important, there is limited attention given to what medicines themselves actually *are*—what is the *thing* that patients are not adhering to or taking optimally?

While anthropologists have credibly highlighted that medicines are not just chemical objects, their “chemical lives” should nonetheless feature in a holistic analysis of medicines themselves as they are often implicated in how they are used and adhered to. Formulation science has repeatedly demonstrated that the physical properties of medicines (their size, shape, taste, smell, mode of delivery) are important for how medicines are perceived and used by patients. In their review of formulation challenges for pediatric practice, for example, Nunn and Williams (2005) note the importance of masking the naturally bitter taste of medicines to encourage adherence in children. Similarly, through experimental research, Wan et al. (2015) reveal how the shape and color of tablets significantly affects patients' perceptions of their ease of use and effectiveness, which in turn impacts adherence.

Beyond this, others have highlighted that medicines' ingredients are vital for patients' decisions about their use. This is perhaps most obvious in the case of dietary preferences, and religious and cultural beliefs. In their research on the impact of religious beliefs in medicines use, Eriksson et al. (2013) found that Muslims, Hindus, and Sikhs refused medicinal devices containing porcine and/or bovine derivatives in all but emergency circumstances. While Enoch et al. (2005) suggest that healthcare practitioners should inform patients about medicinal ingredients, they also found that none of the practitioners surveyed knew the correct ingredients of the medicines prescribed, potentially leading patients to their own research and harmful “tinkering” with their prescribed regimes (to quote Mol, 2008). Sattar et al. (2004) outline four case studies in which patients, upon discovering their medicines contained products prohibited by their religion, immediately stopped treatment, leading to relapses in condition.

In the context of the (re)emergence of natural products drug discovery, the active ingredients in new antibiotics may well be products which have historically sat outside of conventional biomedical models (notably here, clay). Opening up the black box of medicines and their chemical and material lives will enable us to grasp how diverse active ingredients are perceived and influence use. For example, will patients accept clay-based poultices as a legitimate medicine for treating wound infections? How will their perceptions affect their use of poultices? How can prescribers best counsel patients to ensure optimal use of innovative novel medicines? These are important questions for scientific researchers, practitioners and policy-makers in AMR, and ones which sociology is well-placed to answer.

## Discussion

In this paper, we have suggested a new direction for sociological research on AMR that examines the social and material life of antimicrobials themselves. Such a focus is pertinent at a time when diverse materials, particularly natural products, are being explored for their potential as new antimicrobial drug candidates. We selected clay as an appropriate example since its antimicrobial potential is well-documented (Williams, 2019) and it has a thriving research community to which sociology has much to offer but has not yet engaged with. For instance, sociology is conspicuously absent from Henry and Matthews Kwong's (2003, p. 354–355) observations on the diversity of disciplinary perspectives on geophagy: “research is conducted by a striking variety of specialists in the fields of primatology, biology, chemistry, mineralogy, parasitology, medicine, nutrition, anthropology, geography and public health.” Henry and Cring (2013) similarly note that interdisciplinarity is vital for deeper research into the social life of clay. More broadly, sociologists have advocated the need to include our research, approaches, theories and methodologies in AMR research. For Lorencatto et al. (2018), sociologists have a key role to play in supporting prescribing behavior change interventions while Will (2018) argues that more nuanced theorizing around AMR will support sociology to become part of the policy and practice solution.

Taking the illustration of antibacterial clay, we have suggested a number of approaches for sociologists to begin exploring the social and material life of natural antimicrobials. We have mapped these theoretical lenses onto the physical and symbolic mobility of clay into, within, and out of western biomedical laboratories. This is not an exhaustive list of theoretical “ways in” to explore the (re)emergence of natural products in antimicrobial research; there are certainly additional lenses which would be valuable to employ in tandem that we have not touched upon here. Our goal was not to compile a comprehensive account, but to present what we see as fruitful “ways in” as heuristic devices to stimulate discussion within our discipline, and beyond, as to how we might credibly tackle this new direction in AMR research. Moreover, the “ways in” that we have proposed here also have currency for exploring other natural product-based medicines more broadly (i.e., not limited to antimicrobials). The “ways in” we have presented are deliberately disparate to both draw out the diversity of issues enmeshed in questions of natural antimicrobials, and to demonstrate that sociology has the broad theoretical arsenal to approach these. We are not suggesting that any given future research on natural antimicrobials should attempt to synthesize and incorporate *all* of these theoretical frameworks but, rather, bring specific frameworks in and out of prominence in addressing particular aspects of natural antimicrobial materiality. We are, then, suggesting these frameworks as “ways in” for sociologists to begin thinking about a materiality approach to antimicrobial products, rather than providing an instructional schema.

Nonetheless, there are several thematic coalescences and points of confluence in the disparate concepts and frameworks outlined above. Most notably, taken together in much the same way we have presented them here, these frameworks and foci provide a holistic theoretical reading of the “story” of natural antimicrobials' movement into and around western biomedicine. In other words, a single aspect of clay's (and other natural antimicrobials') entrance into the western biomedical gaze should not be fully understood in isolation but, rather, should give space to the tangential issues. For example, an examination primarily focused on the development of new markets around clay products (both medical and cosmetic, from a valuographies perspective) ought to nod to the development of evidence underpinning this market (from an STS perspective) which is, in turn, informed by clay's longer history as a therapy (from a post-colonial perspective).

These diverse theoretical frameworks are all, at various times and to varying degrees, underpinned by notions of space, legitimacy, and practice, where the legitimacy of clay shifts (from “alternative” to mainstream) as it moves into diverse spaces (from global south to north) and is practiced upon in diverse ways (through scientific experimentation). For example, post-colonial approaches can help us to illuminate the ways “traditional” uses of clay in the global south are positioned as illegitimate through “colonial gazes” (Henry and Cring, 2013: 186) and STS frameworks can show us how legitimacy of clay is achieved through scientific experimentation. Outside of the laboratory, legitimacy of a medicine or a drug regime is central to its use by patients (Cohen et al., 2001).

This new route in AMR research requires a considerable degree of interdisciplinarity. While we have argued here in favor of a sociological focus on the social and material lives of natural antimicrobials, we are not advocating a partisan approach where sociologists focus exclusively on the analytical frameworks offered by others in our discipline. Indeed, as we have shown, discipline-hopping frameworks such as STS and post-colonial studies need to be at the heart of this new direction. We contend, moreover, that sociologists should not just collaborate with our close disciplinary neighbors (namely anthropology, geography, and psychology). Rather, we ought to develop networks spanning social, biological, physical, and earth sciences to promote a holistic approach to social and material life. Many of the questions at the center of natural antimicrobials (e.g., the nature of the *stuff* itself, its movement into biomedicine and its commercial value) are shared across disciplines and best addressed through collaborative approaches. Working in pre-existing disciplinary silos constrains the degree to which the material life of natural antimicrobials can be fully understood and their practical and clinical utility fully realized. While laboratory scientists are keenly focused on identifying the physical, mineralogical, and chemical nature underpinning clay's antimicrobial action, its usefulness as a western biomedicine is an inherently social and cultural question. These questions concern, among other issues, whether prescribers are convinced by medicines with clay as an active ingredient and whether patients will adhere to clay-based medicines regimes. Similarly, while sociologists can identify an interesting story in clay's mobility, to convincingly take a materiality approach such as we have advocated above requires a degree of engagement with the production of physical and chemical materiality through diverse scientific techniques.

## Author Contributions

KJ led on the preparation of the manuscript with significant conceptual and design input and contribution from GS.

## Conflict of Interest

The authors declare that the research was conducted in the absence of any commercial or financial relationships that could be construed as a potential conflict of interest.
